# The Relation between Cognitive and Emotional Processes in Children and Adolescents with Neurodevelopmental Disorders—A Meta-Analysis

**DOI:** 10.3390/ejihpe13120194

**Published:** 2023-11-29

**Authors:** Cristina Costescu, Adrian Roșan, Carmen David, Lia Cozma, Andrada Calota

**Affiliations:** Special Education Department, Faculty of Psychology and Educational Sciences, Babeș-Bolyai University, 400029 Cluj Napoca, Romania; adrian.rosan@ubbcluj.ro (A.R.); carmen.david@ubbcluj.ro (C.D.); liamaria.cozma@gmail.com (L.C.); andradacalota@gmail.com (A.C.)

**Keywords:** executive functioning, emotion regulation, affective problems, autism spectrum disorders, meta-analysis

## Abstract

Background: Up to 80% of children with autism spectrum disorders (ASDs) have mental health issues—either emotional or behavioral problems. The underlying mechanisms are still unknown, even if emotional regulation (ER) is considered to play a major role in child and adolescent psychopathology. Several studies link the ability to regulate the intensity and quality of emotions with executive functioning. Therefore, we aimed to investigate the association between executive functions (EFs) and ER and affective problems in children with ASD. Methods: This meta-analysis is based on a literature search of peer-reviewed journals from the following databases: Scopus, ProQuest, Ebsco, Science Direct, Springer Link and Clarivate. We analyzed 15 studies that investigated the link between EF, ER or affective problems (APs) in children and adolescents with ASD aged between 2 and 18 y with ASD. To assess the effect size of the relationship between EF and ER, and EF and AP, 15 studies comprising 54 effect sizes were analyzed. Results: Our findings revealed a small effect size regarding the association between EF and ER, *r* = 0.331, *p* = 0.034, and a small effect size regarding the association between EF and AP, *r* = −0.213, *p* = 0.024. No significant moderators were found. The results are presented in regard to the two analyses developed, as well as a short review of the studies included in the meta-analysis. Conclusion: Even if there are several limitations of this study, especially considering the small number of studies included, the results suggest that it is worth considering EF as an underlying mechanism for the appearance of emotional or behavioral problems in children with ASD. These findings have important implications for the development of ASD intervention plans, as well as for increasing awareness among specialists about the importance of executive functions in school adjustment and social functioning.

## 1. Introduction

Executive functions (EFs) represent a set of cognitive processes that help us control our behaviors and emotions to achieve our goals. They predict academic and social success by helping us to select and monitor successful behaviors that facilitate the achievement of our goals when automatic processes are insufficient [1]. These are higher-order cognitive processes supported by core underlying processes, such as working memory, inhibitory control and cognitive flexibility [2]. Several reviews showed that children with neurodevelopmental disorders (NDDs) have difficulties with EFs [3,4] and moreover, a broad executive disfunction is stable across development and is linked with different criteria of diagnosis within autism spectrum disorders (ASDs), such as repetitive behaviors [5,6]. Inhibition and shifting are mainly associated with rigid behaviors, but also other EFs may impact the social adaptative behaviors, emotional adjustment, and well-being [6,7]. The relation between EFs and emotional regulation was widely investigated and can be considered bidirectional. On the one hand, to choose and implement adaptative emotion regulation strategies, individuals need to control the selection process of the strategy and to flexibly switch from one strategy to another [8]. This level of processing involves working memory, for remembering the adaptative emotion regulation strategies, cognitive flexibility for easily switching from one strategy to another and planning to implement all the steps necessary for the chosen strategy [8]. On the other hand, tasks that are designed to assess or involve EFs or effortful control, such as academic tasks or following specific steps to reach one’s goals, can evoke distress and are very likely to influence the performance of the child. This second explanation for the relation between EF and emotion regulation represents a developmentally dynamic view and it can explain mainly what happens in early childhood and can also partly explain poor emotion regulation skills in children with neurodevelopmental disorders [9,10].

For children with ASD the use of maladaptive coping strategies is linked with EF impairments and in combination with their characteristics (i.e., difficulty in identifying social cues, rigid or stereotypical responses, difficulties in understanding others’ perspective) make them more vulnerable to exhibit higher levels of distress [11,12]. Moreover, emotion dysregulation may act as an underlying mechanism in the development of internalizing and externalizing psychopathology [13,14] and is also linked with peer rejection and victimization [15]. Emotion regulation may be defined as the necessary effort needed to control or modify the intensity of the emotional reaction in order to reach one’s goal [16]. Emotions are usually linked with the appraisal of a specific situation and to a behavioral response, whereas moods are considered more general and stable states [17]. In other words, individuals with emotion dysregulation problems may experience emotions more intensely than others and have a hard time returning to a stable emotional state. Both in children and adolescents with ASD emotion dysregulation may be characterized by difficulties in managing emotional responses, such as experiencing intense or prolonged emotional states, problems in shifting from one emotion to another, and struggles in appropriately expressing or suppressing emotions in different situations with different presentation of symptoms at different developmental stages [18]. 

Considering the fact that children with ASD and emotion dysregulation can manifest in various ways, including emotional instability, impulsivity, mood swings, and difficulty in coping with stress or managing interpersonal relationships, this leads to increased peer problems. Another study showed that there is a link between adaptative emotion regulation strategies and quality of social relationships and enhanced ability to understand others’ mental states [19]. Namely, emotion dysregulation can be considered a risk factor for the development of an affective disorder, due to the intense and more frequent emotional and physiological responses [20]. 

This study outlines the current status in the field of executive functions and emotion regulation and affective disorders and takes an important step forward by including existing studies in a quantitative meta-analysis. For the purpose of the this meta-analysis we defined affective disorders as being any group of conditions of mental and behavioral disorders where the main disturbance is in the mood of the person (i.e., depression, anxiety). To ensure appropriate use of intervention techniques regarding EF or ER strategies, professionals must have a clear understanding of the opportunities and challenges such treatments will provide in their daily practice. Moreover, we consider that it is very important to clearly understand the relationship between the two constructs. The specific objectives of this study were to quantify the association between EF and ER or affective problems in children and adolescents with neurodevelopmental disorders by determining the overall effect size of this association (establishing the effect size for specific outcomes). Moreover, an exploratory approach was taken for reviewing moderators’ impact and no specific hypotheses were made.

## 2. Materials and Methods

### 2.1. Literature Search and Selection of Studies

**Figure 1:**
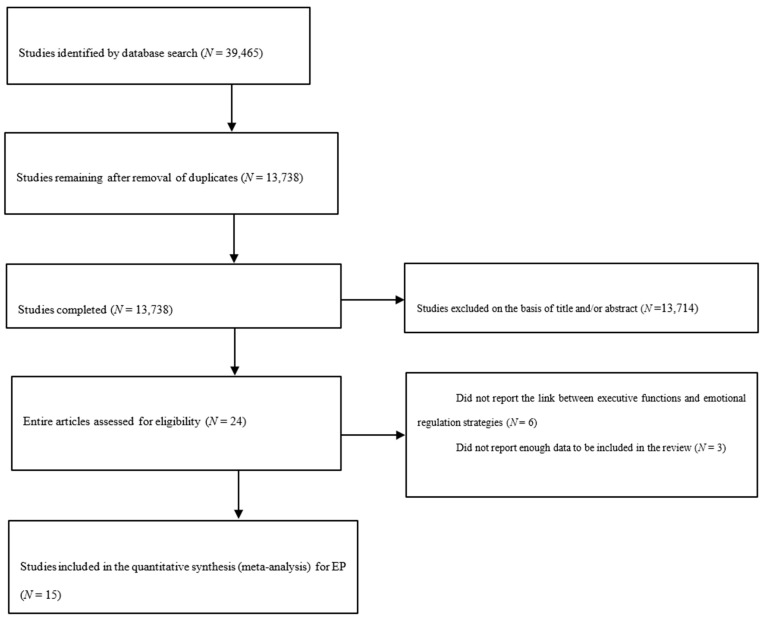
The PRISMA Diagram.

We conducted a literature search, focused on intervention studies published between 1990 and 2022 in the databases Scopus, Proquest, Ebsco, Science Direct, Springer Link and Clarivate. A protocol was performed prior to the meta-analysis but was not included in the PROSPERO database. To be included in this review, studies must have met the following criteria: (a) no single case studies; (b) link between executive functions and affective problems; (c) to measure executive functions and emotional regulation strategies; (d) to be published only in English; (e) peer-reviewed only; (f) between 1990 and 2022; (g) no intervention; (h) participants must be between 2 and 18 years old. Inclusion criteria was assessed by two independent researchers which had an 80% agreement between them for study inclusion, with the other 20% discussed with the research team. Based on these criteria, we included a total of 15 studies. The PRISMA flow chart of this selection process is described in Figure 1.

We used the following search terms: (“executive function” OR “executive functioning” OR “executive control” OR “executive attention”) AND (“emotion regulation” OR “ emotion control” OR “emotion competence” OR “emotion development”) AND (“autism” OR “autism disorder” OR “autism spectrum disorder”); (“attention control” OR “attention shifting” OR “working memory”) AND (“emotion regulation” OR “emotion control” OR “emotion competence” OR “emotion development”) AND (“autism” OR “autism disorder” OR “autism spectrum disorder”); (“inhibition” OR “planning” OR “monitoring”) AND (“emotion regulation” OR “emotion control” OR “emotion competence” OR “emotion development”) AND (“autism” OR “autism disorder” OR “autism spectrum disorder”); (cognitive flexibility OR problem solving OR effortful control) AND(“emotion regulation” OR “emotion control” OR “emotion competence” OR “emotion development”) AND (“autism” OR “autism disorder” OR “autism spectrum disorder”) and because in Science Direct we were restricted by the number of characters, we had (“inhibition” OR “planning” OR “monitoring”) AND (“autism”) AND (“emotion regulation” OR “emotion control” OR “emotion competence” OR “emotion development”) AND (“autism”); (“executive function” OR “executive functioning” OR “executive control” OR “executive attention”) AND (“emotion regulation” OR “ emotion control” OR “emotion competence” OR “emotion development”) AND (“autism”); (“attention control” OR “attention shifting” OR” working memory”) AND (“emotion regulation” OR “emotion control” OR “emotion competence” OR “emotion development”) AND (“autism”); (cognitive flexibility OR problem solving OR effortful control) AND (“emotion regulation” OR “emotion control” OR “emotion competence” OR “emotion development”) AND (“autism”). 

The search strategy produced a total of 39,465 recordings. After removing duplicates and irrelevant entries, a total of 13,738 full-text articles remained to be assessed for eligibility. 

### 2.2. Data Extraction

For each of the included studies we extracted the following information: identification data (author, year of publication), outcome, effect size data, and several moderator variables, including methodological characteristics of the study and sample characteristics. We grouped the specific outcome measures based on several independent criteria: (a) type of variable (emotion regulation, executive function, and emotional problems (b) type of measurement (self-report and parent report). In terms of the methodological characteristics of the study, we grouped studies in (a) comorbidities (b) ASD score (c) IQ and (d) age. Data were extracted independently by two of the authors. Both researchers extracted the study’s name, the number of participants for each study, the characteristics of the population, the description of the tasks, the outcomes and the correlation coefficients. Coding of individual outcomes was performed by the two independent researchers and verified by the first author. In 80% of the cases there was a consensus between the researches; where there were discrepancies each study was discussed independently. Data extracted are presented in Table 1.

### 2.3. Data Meta-Analysis

For the meta-analysis we used the program Comprehensive meta-analysis where we introduced 58 data sets from 15 studies which represent correlations between either executive functions (EFs) and affective problems (APs) or EF and emotion regulation (ER). Further we ran two analyses, one for the correlations between EF and AP and one for the correlations between EF and ER. For both of the meta-analyses we used the weighted mean of the outcomes, and we selected the random model because we do not assume that all studies share the same effect size. Heterogeneity was assessed using tau squared. The meta-analysis between EF and AP was realized on nine studies and the analysis between EF and AP was realized on eight studies. In addition, for us to be able to integrate the correlation coefficients we ran a Fisher transformation. The transformation converts the skewed and bounded sampling distribution of r into a normal distribution for z.

We also wanted to see if age, IQ and the measure type of EF moderate the correlations. Others found age as a relevant moderator for EF in children with ASD. In addition, IQ moderates interference inhibition performance in children with ASD [21]. Moreover, most studies looked at different subcomponents of EF and ER, thus we are not expecting homogeneity across the studies. So, we ran moderation analyses to see if the specified variables moderated the relation between EF and ER, and between EF and AP.

**Table 1:** The studies included in the meta-analysis with the measures included.

Study	N	Type of Population Age	Autism Severity IQ	Tasks Description	Measured Outcome	Pearson Correlation
Andersen, Skogli, Hovik, Egeland and Oie (2015) [22]	79	ASD + TD 9–16 years old	HFA IQ = 98.5	Short Moods and Feelings questionnaire Child Behavior Checklist Inhibition: Color—Word Interference Test, Condition 3 Cognitive Flexibility: Color—Word Interference Test, Condition 4 Working memory: Letter—Number Sequencing Test	Depression Symptoms Emotion regulation Inhibition Cognitive flexibility Working memory	*r* = −0.08 (depression + working memory) *r* = −0.35 (depression + inhibition) *r* = −0.23 (depression + cognitive flexibility) *r* = 0.14 (affect + working memory) *r* = −0.04 (affect + inhibition) *r* = −0.07 (affect + cognitive flexibility)
DeLucia, McKenna, Andrzejewski, Valentino and McDonnell (2021) [23]	4	ASD + TD 4–6 years old		Day/Night Task Emotion Regulation Checklist Strengths and Difficulties Questionnaire	Inhibitory control Emotion regulation facets Internalizing problems	*r* = 0.03 (ER+ inhibitory control) *r* = −0.6 (internalizing problems + inhibitory control) *r* = −0.32 (lability/negativity + inhibitory control)
Fernandez-Prieto, Moreira, Cruz, Campos, Martínez-Regueiro, Taboada, Carracedo and Sampaio (2020) [24]	79	ASD 4–16 years old		Child Behaviour Checklist Executive functioning domains derived from CBCL	Anxious, withdrawn/depressed. Working memory	*r* = −0.11 (anxious/depressed+ working memory) *r* = 0.07 (withdrwan/depressed+ working memory)
Goldsmith and Kelley (2018) [25]	145	ASD 5–17 years old	IQ = 84.5	Emotion Regulation Questionnaire Autism Quotient	Reappraisal/suppression Attention switching	*r* = −0.24 (reappraisal +attention switching) *r* = −0.38 (suppression + attention switching)
Hollocks, Jones, Pickles, Baird, Happé, Charman, and Simonoff (2014) [26]	90	ASD 14–16 years old		Strengths and Difficulties Questionnaire Opposite worlds Trail making Numbers backwards Card sorting task	Emotional symptoms Inhibition Attentional switching Working memory Shifting	*r* = −0.34 (anxiety + attention switching) *r* = −0.05 (derpession + attention switching) *r* = −0.24 (cognitive set shifting+ anxiety) *r* = −0.23 (cognitive set shifting+ depression) *r* = −0.23 (interference inhibtion + a nxiety) *r* = −0.09 (interference inhibtion+ depression) *r* = −0.10 (working memory+ anxiety) *r* = −0.01 (working memory+ depression)
Jahromi, Bryce and Swanson (2013) [27]	40	ASD + TD Age: *M* = 54.57 months, *SD* = 11.31 months		Emotion Regulation Checklist The Day/Night Task Behavior Rating Inventory of Executive Function-Preschool Version Child Behavior Questionnaire–Short Form	Emotion regulation Inhibition Inhibitory control Effortful control	*r* = 0.48 (ER + effortful control) *r* = 0.82 (ER + executive function composite)
Guy, Souders, Bradstreet, DeLussey and Herrington (2014) [28]	36	ASD + TD Age: 12.27 years (ASD)/13.12 years (TD)		Behavior Rating Inventory of Executive Function Child Anxiety Related Disorders	Shifting Anxiety	*r* = 0.62 (anxiety + shifting)
Tajik-Parvinchi, Farmus, Modica, Cribbie and Weiss (2021) [20]	48	ASD + ADHD+ LD + CP 8–13 years old	IQ = 104,69	Behavior Rating Inventory of Executive Function Emotion Regulation Checklist Behavior Assessment System for Children	Inhibition, Working memory, Shifting Emotion regulation Lability/negativity	*r* = 0.27 (inhibition + internlizing) *r* = 0.35 (working memory+ internalizing) *r* = 0.27 (shifting + internalizing) *r* = −0.23 (inhibition + emotion regulation) *r* = −0.07 (working memory+ emotion regulation) *r* = −0.28 (shiftting + emotion regulateon) *r* = 0.50 (inhibition + lability/negativity) *r* = 0.43*(working memory+ lability/negativity) *r* = 0.35 (shiftting + lability/negativity)
Lawson, Papadakis, Higginson, Barnett, Wills, Strang, Wallace and Kenworthy (2014) [29]	125	ASD + ADHD 6–16 years old	IQ = 109	Behavior Rating Inventory of Executive Function Child Behavior Checklist	Shifting, inhibition Anxiety/depression	*r* = 0.17 (anxious/depressed+ inhibition) *r* = 0.39 (anxious/depressed+ shiftting)
Lieb and Bohnert (2017) [30]	127	ASD + Asperger’s, + Pervasive Developmetal Disorder Not Otherwise Specified 12–17 years old		Behavior Rating Inventory of Executive Function Achenbach Child Behavior Checklist—Depression Scale Achenbach Youth Self Report—Depression Scale	Inhibition, Working memory and Shifting Depression	*r* = 0.36 (inhibition + depression) *r* = 0.46 (shiftting + depression) *r* = 0.5 (working memory +depression) *r* = 0.21 (inhibiton + depression -YSR) *r* = 0.34 (shiftting + depression–YSR) *r* = 0.34 (working memory + depression (YSR)
Ozsivadjian, Hollocks, Magiati, Happe, Baird and Absoud (2021) [31]	95	ASD 5–18 years old	IQ = 98.5	Revised Child Anxiety and Depression Scale Strengths and Difficulties Questionnaire Flexibility Scale-Revised	Anxiety, Depression Emotion problems Cognitive inflexibility	*r* = 0.39 (cognitive inflexibility + anxiety/depression) *r* = 0.34 (cognitive inflexibility + emotion problems)
Ros and Graziano (2019) [32]	100	ASD + ADHD +TD Age: m = 4.73 years		Behavior Rating Inventory of Executive Functions-Preschool Version Head-Toes-Knees-Shoulders Task Laboratory Temperament Assesment Battery	Executive functioning Emotion regulation	*r* = 0.08 (EF—parent report + global regulation) *r* = 0.19 (EF—tearcher report + global regulation) *r* = 0.67 (EF—parent report + ER parent report) *r* = 0.35 (EF—teacher report + ER teacher report) *r* = 0.27 (EF—parent report + ER—teacher report) *r* = 0.30 (EF—teacher report + ER parent report) *r* = −0.21 (EF performance + ER parents report) *r* = −0.05 (EF performance + ER teacher report) *r* = −0.07 (EF performance + global regulation)
Rohr, Kamal and Bray (2019) [33]	276	ASD + TD 8–13 years old	IQ = 114.35	Behavior Rating Inventory of Executive Function	Emotion control, Inhibition, Shifting	*r* = 0.61 (inhibition + emotion control) *r* = 0.79 (shiftting + emotion control)
Jahromi, Chen, Dakopolos and Chorneau (2019) [34]	38	ASD + TD 3–6 years old		Emotion Regulation Checklist Day/Night task Hand Game task	Emotion regulation Inhibition (two types of tasks)	*r* = 0.24 (inhibition D/N + ER) *r* = 0.22 (inhibition HGT + ER)
Hutchison, Müller and Iarocci (2019) [35]	186	ASD + TD 6–13 years old		Behavior Rating Inventory of Executive Function	Emotion control, Shifting and Emotion regulation	*r* = 0.55 (inhibition + emotion control) *r* = 0.68 (shifting + emotion control)

Note: N = number of participants; ASD = Autism Spectrum Disorder; TD = typical development, HFA = High Functioning Autism; r = correlation coefficient; CBCL = Child Behavior Checklist.

## 3. Results

The results related to variability between studies show a low-to-moderate heterogenety, Tau Squared = 0.210, *Q* = 1000.120, *I*_2_ = 94.301.

These results indicate that higher scores for executive functions are associated with lower scores for affective problems for children with ASD. This means that children that have fewer executive functions deficits will have less affective problems. The overall effect size between the two variables is rather small. There can be seen a statistically significant negative relation *r* = −0.213, 95%, CI [−0.384, −0.028], *p* = 0.024, between EF and AP. We assumed that [31] is an outlier on the basis that its CI does not overlap at all with the overall effect size CI. If we exclude the outlier from the analysis, it shows a small increase in the overall effect size *r* = −0.284, 95% CI [−0.393, −0.168], with a *p* value = 0.000. Results are shown in Table 2 and Table 3.

For the next analysis, we looked at the relationship between EF and ER and it was run on eight studies. The results show a small effect size regarding the association between EF and ER, *r* = 0.331, 95% CI [0.025, 0.581], with a *p* value = 0.034. This indicates that higher scores for EF are somewhat associated with higher scores for ER strategies in children with ASD. As in the previous analysis, we assumed that [33] is an outlier on the basis that its CI does not overlap at all with the overall effect size CI. After we exclude the outlier from the analysis, it shows a decrease in the overall effect size *r* = 0.250, 95% CI [−0.005, 0.474], with a *p* value = 0.054, which shows an insignificant correlation. Results can be seen in Table 4 and Table 5.

### 3.1. Funnel Plots

**Figure 2:**
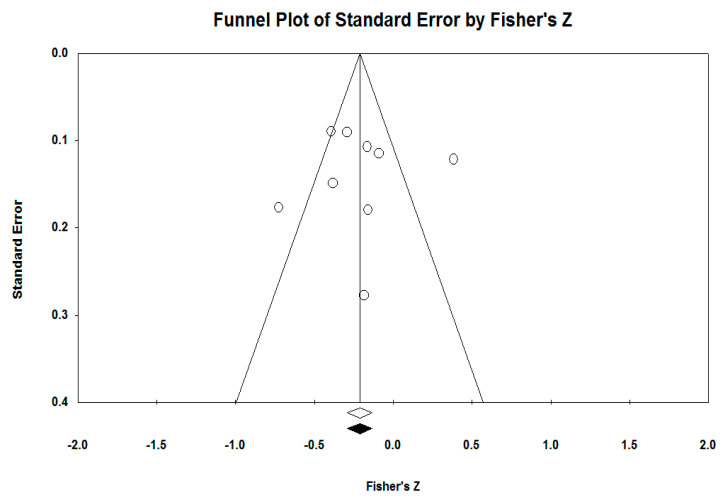
Funnel plot affective problems.

The first funnel plot (Figure 2) analyzed is the one for the relationship between EF and AP. The Egger regression test is statistically insignificant (intercept −0.08, 95% CI [−6.38, 6.20], *t* = 0.03, *df* = 7, *p* = 0.97). The result suggests an asymmetrical distribution. We used the trim and fill method of Duval and Tweedie to find out the number of studies that are missing. The method showed that there are no studies missing to the left of the mean effect. The estimate of the displayed points based on the random effects model is *d* = −0.21, 95% CI [−0.28, −0.13], and after using the trim and fill method the values have not changed due to not adding any other study.

The nine studies obtained *Z* = −4.99, *p* < 0.00, then we used Fail-safe N to see how many studies would be needed for the analyses to have statistical power. The calculation showed that 50 more studies are needed.

**Figure 3:**
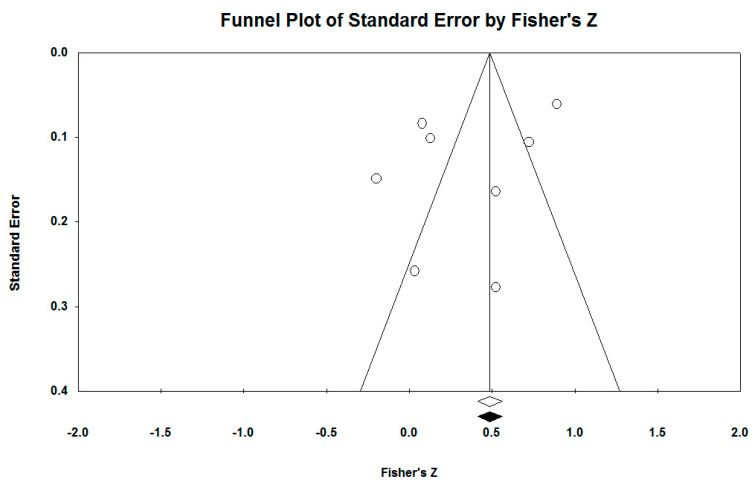
Funnel plot emotion regulation.

The second funnel plot (Figure 3)analyzed is for the relation between EF and ER. The Egger regression test is statistically insignificant (intercept −3.78, 95% CI [−11.47, 3.91], *t* = 1.20, *df* = 6, *p* = 0.27). The result suggests an asymmetrical distribution. We used the trim and fill method of Duval and Tweedie to find out the number of studies that are missing. The method suggests that there are no studies missing. The estimate of the displayed points based on the random effects model is *d* = 0.45, 95% CI [0.39, 0.50], and after using the trim and fill method the values have not changed. The transformation converts the skewed and bounded sampling distribution of *r* into a normal distribution for *Z*. The eight studies obtained *Z* = 1.95, *p* < 0.00, then we used Fail-safe N to see how many studies would be needed for the analyses to have statistical power *p* = 0.05. It showed that 188 studies are needed.

### 3.2. Moderators

Regarding the relation between EF and ER, age group does not have a significant effect as a moderator. The group age 2–6 y is the only one to have a significant moderator relation, but it has only a small correlation. The 6–12 y age group has a bigger correlation than the 2–6 y age group, but is not statistically significant. This difference can be explained by the ability of older children to rely more on themselves for emotional regulation, rather than on external factors. As it can be seen from Table 6, the 12–18 y age group has a smaller relation between EF and ER and although this is the case, due to the fact that this group is represented by only one study, we cannot draw any further conclusions on its effect. Intelligence scores have no moderation effect either. Although the difference between the 70–90 y and 90–110 y age groups is obvious, the results are inconclusive due to the high variability of the 90–110 y age group score. The moderate effect of the unspecified group leads us to the conclusion that there might be a hidden moderation effect. Regarding measurement type, it did not have a moderation effect, but the indirect measure group showed a stronger correlation and the only one that was statistically significant.

The same moderators have been analyzed with concern to the relation between EF and AP (see Table 6). The first moderator analyzed was age group. There is no moderation effect and only the 12–18 y age group show a significant correlation between EF and AP. The effect size for this group is also bigger which may be explained by the fact that, as children grow older, they develop more internal mechanisms of soothing. IQ level also had no moderation effect, but groups with higher IQ (90–110) displayed a slightly stronger correlation. IQ and EF do overlap in a certain way, so we expect IQ to have some effect on AP. Measurement type did not have a moderation effect, nor did any group have any significant result.

## 4. Discussion

Emotional dysregulation influences the majority of psychiatric disorders in children and adolescents with or without ASD. However, the way ER strategies are being selected and used is still unknown and even if those strategies are essential they are often neglected by professionals when the intervention plans are developed. All the studies included in our meta-analysis try to investigate this relationship between EF and ER or how EF influences the appearance of AP. The main assumption is that the cognitive processes (i.e., attention switching, inhibition, cognitive flexibility, working memory) interact with reactive processes. Our results indicate that higher scores for executive functions are associated with lower scores for affective problems for children with ASD. The overall effect size between the two variables is rather small. When it comes to the link between EF and ER, similar results were obtained. The findings showed significant, but small effect size regarding the association between EF and ER. This indicates that higher scores for EF are somewhat associated with higher scores for the use of more adaptative ER strategies in children with NDD. In the following section we will briefly describe the studies included in the meta-analysis.

Tajik–Parvinchi et al. [20] included 48 children in their study aged between 8 and 13 years diagnosed with ASD, ADHD, cerebral palsy and learning disabilities and measured the relationship between working memory, inhibition, shifting and internalizing and externalizing symptoms. They reached the conclusion that children with greater cognitive challenges use maladaptive emotion regulation strategies when they go through a stressful event. Moreover, the basic executive functions predicted emotional dysregulation, which was a significant predictor for both internalizing and externalizing problems. 

De Lucia et al. [23] investigated emotional and behavioral self-regulation, including lability and inhibitory control in a sample of 45 preschoolers with and without ASD. They used direct measurements for inhibitory control (such as day/night task) and other reports for measuring emotion regulation and emotional lability and children internalizing and externalizing problems. Their findings suggest that emotional lability is associated with both emotional and behavioral problems, whereas the ability to apply emotion regulation may act as a protective factor against emotional problems for children that have high levels of autistic traits. Moreover, as a factor that can influence children’s abilities, the authors investigated maternal rigidity and maternal pragmatic language, which were significantly associated with inhibitory control, with respect to emotional regulation and higher child negativity. These last findings should be considered exploratory, and more studies are needed to confirm the relation between the constructs. 

Guy et al. [28] aim in their study to examine the respiratory sinus arrhythmia (RSA) in school-aged children in relation to executive functioning, anxiety and adaptative socialization skills. Among their primary outcomes, showing that RSA is decreased in children with ASD compared to a control group with typically developing children, they also found an interesting results regarding anxiety and EFs. According to the information revealed by the parents (the measurements used were parent-based questionnaires) the ability to shift from one set of rules to another and to accept changes in the environments is strongly associated with anxiety. Even if they tested only 19 children with ASD, and 22 typically developing children, their data support the used of RSA as a biomarker for ER deficits in ASD and its link with anxiety-related disorders and lower socialization skills. 

Hollocks et al. [26] investigated the association between executive functioning and anxiety and depression in adolescents with ASD aged between 14 and 16 years. Besides the above-mentioned variables, they also investigated social cognition by using several standardized measurements. Their results showed that difficulties in executive functioning was significantly related to anxiety symptoms in adolescents with ASD, but not with depressive symptoms. Moreover, no relation was found between social cognition and affective symptoms. Several studies have previously suggested a link between social functioning and understanding and anxiety disorders in people with ASD [36]. However, even if they used four well-known and validated tasks for social cognition this relation was not significant. One possible explanation is the fact that it is very difficult to separate the overall ASD symptoms and their impact on affective problems from social cognition difficulties.

Jahromi, Bryce and Swanson [27] included 40 children in their study, 20 with high-functioning autism and their peers. Apart from EF and ER, they also measured prosocial peer engagement, joint engagement during parent–child interaction and effortful control. Their results showed that EF predicted both emotional and behavioral school engagement, whereas ER predicted peer engagement. Effortful control was correlated with joint engagement, executive functions and ER. The composite score of EF measured with a standardized other report scale was associated with ER; in addition, the performance in the day/night task was positively associated with ER. The authors mention that EF explained the most variability in ER and was the “most salient contributor to both behavioral and emotional school engagement” (p. 243). 

The group of Jahromi and his colleagues [34] also examined the link between delay of gratification and ER, EF, effortful control and joint attention. In terms of the performances in the delay-of-gratification task, their findings revealed that preschoolers with ASD waited for a shorter duration compared to typically developing children and expressed less positive affect during the task and more temptation-focused behaviors, as strategies while waiting. ER was found to have the strongest association with children’s temptation-focused strategies, suggesting that delay of gratification may also include the capacity to cope with intense emotions. Their results did not reveal significant association between ER and the performance in a hand game, nor between ER and the performance in the day/night game (both games measure inhibition). On the other hand ER was strongly correlated with effortful control. 

Lawson et al. [29] included 125 children in their study, 70 of them with an ASD diagnosis and 55 with ADHD and aged between 6 and 16 y. They used parent report questionnaire to measure EF, anxiety/depression and aggressive behaviors. Their findings showed that children with ADHD tend to have more inhibition difficulties and comorbid aggressive and oppositional behaviors, while children with ASD have more difficulties in cognitive flexibility which is associated with anxiety and depression. Therefore, they consider parent-reported inflexibility and disinhibition as crucial components of ASD and ADHD that may contribute to psychiatric symptoms in this population. They also found that inhibition scores were positively associated with aggressive behaviors, whereas shifting scores were correlated with anxiety/depression scores, and no significant correlation was found between shifting and anxiety/depression. 

Leib and Bohnert [30] evaluated association between several EF, social impairment, friendship quality and depressive symptoms/loneliness in 127 high-functioning ASD adolescents. They found significant associations between inhibition, shifting, working memory and self- and other-reported depression. Another interesting result is the fact that the relation between EF and adjustment is mediated by social impartment. However, this was not the case for friendship quality. Since EFs are associated with social impairments, the authors consider having a better understanding of it—and addressing a person’s EF may be an important component of a successful intervention for adolescents with ASD. 

Ozsivadjan et al. [31] tried to investigate in their study the underlying cognitive mechanisms for externalizing and internalizing difficulties in ASD. They considered in their study cognitive inflexibility, intolerance of uncertainty and alexithymia. Their sample consisted of 95 children and adolescents aged between 5 and 18 years with ASD. Cognitive inflexibility was significantly associated with both depressive symptoms and emotional problems. It played a direct role in ASD symptoms and behavioral problems, and an indirect rol—via intolerance of uncertainty—in emotional problems. In addition, out of the three underlying mechanisms investigated, only cognitive inflexibility predicted significantly externalizing symptoms. 

Rohr, Kamal and Bray [33] examined whole-brain functional correlations and behavioral regulation through connectome predictive modeling. They analyzed 276 children with or without autism aged between 8 and 13 years of age. They identified networks whose functional correlations predicted individual differences in emotional and behavior regulation. From their behavioral measurements a significant relation between emotional control and shifting and inhibition emerged. Their study highlights the possibilities for understanding how the brain’s functional organization may be associated with cognitive and behavioral difficulties in children with ASD or other neurodevelopmental disorders. 

Goldsmith and Kelley [25] investigated the relation between emotion regulation and ASD symptomatology on 145 youths with ASD. For measuring ER, they used an emotion regulation auestionnaire [37] which allowed them to access different types of cognitive strategies, even if the authors modified each item to be suitable for parent-reporting. Their results showed that the ability of attention-switching is significantly correlated to reappraisal. Moreover, the use of more adaptative emotion regulation strategies, such as reappraisal, predict fewer social impairments. 

Anderson et al. [22] enrolled 34 children with high-functioning autism and 45 typically developing children and assessed cognitive flexibility, inhibition and working memory using standardized performance tasks applied to children and emotional functioning through a self-report depression symptoms questionnaire and parent-rated emotional functioning. They did not find significant associations between any of the EF measured and emotional problems or depressive symptoms. They made measurements in T1 and after two years using the same instruments. Their findings suggested that depressive symptoms decreased over time, ASD severity was stable, and EF improved. 

Fernandez-Prieto et al. [24] focused mainly on the link between sensory processing behaviors, executive functioning and behavior and emotional problems in 79 children and adolescents with ASD. Even though the specific association between depression/withdrawn or affect problems and working memory was not significant, using structural equation modeling methods they observed a mediating effect of the executive functioning in the relation between sensory processing and behavioral problems. They also found that the ER difficulties are highly associated with behavioral problems and that working memory difficulties were associated with repetitive/obsessive and aggressive behaviors. 

Ros and Graziano [32] aim to identify the profiles of self-regulation across executive functioning and ER and examine the impact on the intervention outcome in preschoolers with ASD, ADHD and typically developing children. They used both parent and teacher reports and several tasks to assess EF and ER. In terms of intervention outcomes, they measured school readiness and externalizing behavior problems. In terms of the profiles identified, the study revealed the following: low ER and EF difficulties, high ER deficits, high RF deficits and moderate ER and EF deficits. According to their findings, symptoms of ASD were predictive within the high EF deficits, while symptoms of ADHD were predictive within high ER deficits. They also reported a significant association between EF and ER, but no link between EF and global regulation, in either parent- or teacher-reports. 

Hutchinson, Muller and Iarocci [35] compared a group of 92 children with ASD with 94 typically developing children in terms of their executive functioning, verbal conversations and functional communication. There were no significant differences between the two groups for age and IQ. As expected, children with ASD showed more EF deficits than typically developing children. However, for both groups the authors revealed that metacognition represents a strong factor for functional communication, while behavioral regulation and inhibition predict verbal conversational skills. Also, another study found that IQ moderates interference inhibition performance in children with ASD [38].

All the above-analyzed studies have mainly the same outcome, the same relation between EF and ER or relation between EF and AP. However, the types of measurements used (direct performance measurements, self-report/parent/teacher report questionnaires) make the data collected heterogeneous. Moreover, in order to have a better idea of this relationship at different ages, we included children from 2-to-18 years old; however, the ability of executive functioning and ER is very different in the developmental stages and an analysis based on age groups was not possible due to the small number of studies. The majority of the analyzed studies also investigated other types of variables, such as school adjustment, loneliness, and intolerance of uncertainty. Unfortunately, all these variables were not considered for our analysis because they were not within the scope of our research.

## 5. Conclusions

Emotion dysregulation is a relatively new concept in the domain of ASD and specific interventions are still under development. Our main goal was to increase awareness about the importance of considering executive functioning when such treatments are being developed. Regardless of their age, the programs for children with ASD, and also with other neurodevelopmental disorders, need to include at least one module that trains their executive functioning and teaches them to use adaptative ER strategies. Even if we found only a small effect size, due to the small number of studies investigated and to their increased heterogeneity, our results emphasize the link between the cognitive and emotional processes. In other words, our study shows that executive functioning is linked with the use of adaptative emotion regulation strategies. On the one hand it can be linked with the process of choosing and implementing adaptative emotion regulation strategies, because children and adolescents need to control which strategy they choose and then flexibly switch from one strategy to another [8]. On the other hand, anxiety or depression symptoms can interfere with the executive functioning, leading to impaired capacity of paying attention, remembering and planning. 

There are several limitations of our study. Firstly, considering the fact that all the variables investigated are quite large constructs, all of the have subcomponents and all these different subcomponents were analyzed together under the same big construct and make it much more difficult to interpret the results. Future studies should analyze each subcomponent and provide a more detailed analysis on each. Secondly, the small number of studies is one of the most important limits of this research. Most results do not reach significance although they have a small–medium correlation size, and, a big difference in correlations between groups did not lead to a moderation effect. This can be attributed to low statistical power which is directly affected by the number of studies and number of participants. There was evidence of a high level of heterogeneity, as shown by the following indicators, *Q* (57) = 1000.120, *p* < 0.000 *I*_2_ = 94.310; in this case we analyzed whether one of the potential moderator variables could have explained the heterogeneity found on the overall effects. Since the moderators analyzed could not explain the heterogeneity of the results, there must be other moderating factors that have not been discussed and analyzed or are not known that explain the heterogeneity of the results.Thirdly, even if the majority of the groups that were included and analyzed in this meta-analysis are children with ASD, some of the articles included in their analysis children with comorbidities or with other neurodevelopmental disorders. Therefore, some of the conclusions drawn from this meta-analysis could apply for different types of developmental disorders, not exclusively to children with ASD. 

Our work sheds some light on an important topic in special education and in psychology: the link between executive functioning and emotional dysregulation and AP. This relationship will help to better understand the underlying mechanism of the internalization and externalization problems that may appear in children and adolescents with neurodevelopmental disorders. Moreover, it can contribute to the development of the next generation of screening, diagnostic and intervention approaches and programs. 

## Figures and Tables

**Table 2:** Correlation between EF and AP.

Affective Problems						
Study	Outcome	Statistics for Each Study				
		Correlation	Lower Limit	Upper Limit	Z-Value	*p*-Value
Guy (2014) [28]	Anxiety + EF	−0.620	−0.790	−9.361	−4.101	0.000
DeLucia (2021) [23]	Combined	−0.183	−0.622	0.344	−0.666	0.505
Tajik-Parvinchi (2021) [20]	Combined	−0.365	−0.588	−0.090	−2.565	0.010
Lieb (2017) [30]	Combined	−0.374	−0.515	−0.214	−4.377	0.000
Andersen (2015) [22]	Combined	−0.158	−0.471	0.191	−0.885	0.376
Lawson (2014) [29]	Combined	−0.284	−0.438	−0.114	−3.222	0.001
Hollocks (2014) [26]	Combined	−0.136	−0.358	0.045	−1.537	0.124
Fernandez-Prieto (2020) [24]	Combined	−0.090	−0.305	0.134	−0.787	0.431
Ozsivadjian (2020) [31]	Combined	0.365	0.143	0.552	3.145	0.002
		−0.213	−0.384	−0.028	−2.251	0.024

**Table 3:** Correlation between EF and AP without [31].

Affective Problems						
Study	Outcome	Statistics for Each Study				
		Correlation	Lower Limit	Upper Limit	Z-Value	*p*-Value
Guy (2014) [28]	Anxiety + EF	−0.620	−0.790	−9.361	−4.101	0.000
DeLucia (2021) [23]	Combined	−0.183	−0.622	0.344	−0.666	0.505
Tajik-Parvinchi (2021) [20]	Combined	−0.365	−0.588	−0.090	−2.565	0.010
Lieb (2017) [30]	Combined	−0.374	−0.515	−0.214	−4.377	0.000
Andersen (2015) [22]	Combined	−0.158	−0.471	0.191	−0.885	0.376
Lawson (2014) [29]	Combined	−0.284	−0.438	−0.114	−3.222	0.001
Hollocks (2014) [26]	Combined	−0.136	−0.358	0.045	−1.537	0.124
Fernandez-Prieto (2020) [24]	Combined	−0.090	−0.305	0.134	−0.787	0.431
		−0.284	−0.393	−0.168	−4.765	0.000

**Table 4:** Correlation between EF and ER.

Emotion Regulation						
Study	Outcome	Statistics for Each Study				
		Correlation	Lower Limit	Upper Limit	Z-Value	*p*-Value
Tajik-Parvinchi (2021) [20]	Combined	−0.195	−0.454	0.095	−1.324	0.186
Jahromi (2019) [34]	Combined	0.033	−0.441	0.492	0.129	0.898
Goldsmith (2018) [25]	Combined	0.077	−0.087	0.237	0.925	0.355
Ros (2019) [32]	Combined	0.128	−0.070	0.316	1.266	0.206
DeLucia (2021) [23]	ER + inhibitory control	0.480	−0.021	0.788	1.886	0.059
Jahromi (2013) [27]	ER + effortful control	0.480	0.198	0.689	3.181	0.001
Hutchison (2019) [35]	Combined	0.619	0.475	0.731	6.828	0.000
Rohr (2019) [33]	Combined	0.711	0.647	0.766	14.546	0.000
		0.331	0.025	0.581	2.116	0.034

**Table 5:** Correlation between EF and ER without [33].

Emotion Regulation						
Study	Outcome	Statistics for Each Study				
		Correlation	Lower Limit	Upper Limit	Z-Value	*p*-Value
Tajik-Parvinchi(2021) [20]	Combined	−0.195	−0.454	0.095	−1.324	0.186
Jahromi(2019) [34]	Combined	0.033	−0.441	0.492	0.129	0.898
Ros(2019) [32]	Combined	0.128	−0.070	0.316	1.266	0.206
Goldsmith(2018) [25]	Combined	0.077	−0.087	0.237	0.925	0.355
Jahromi(2013) [27]	ER + effortful control	0.480	0.198	0.689	3.181	0.001
Hutchison(2019) [35]	Combined	0.619	0.475	0.731	6.828	0.000
DeLucia(2021) [23]	ER + inhibitory control	0.480	−0.021	0.788	1.886	0.059
		0.250	−0.005	0.474	1.924	0.054

**Table 6:** Moderators for EF + ER and for EF + AP.

	Moderator	Category	K	R	CI	Qb	*p*
EF + ER	Age	2–6	4	0.267	[0.03, 0.48]	3.290	0.193
		6–12	3	0.452	[−0.05, 0.77]		
		12–18	1	0.077	[−0.08, 0.23]		
	IQ	70–90	1	0.128	[−0.07, 0.31]	1.727	0.422
		90–110	2	0.341	[−0.61, 0.89]		
		Unspecified	5	0.365	[0.04, 0.61]		
	Measure type (EF)	Direct	3	0.083	[−0.26, 0.41]	1.370	0.242
		Indirect	6	0.365	[0.02, 0.62]		
EF + AP	Age	2–6	1	−0.183	[−0.62, 0.34]	2.351	0.309
		6–12	5	−0.105	[−0.35, 0.16]		
		12–18	3	−0.377	[−0.58, −0.13]		
	IQ	70–90	1	−0.163	[−0.35, 0.04]	1.062	0.588
		90–110	3	−0.284	[−0.40, −0.15]		
		Unspecified	5	−0.192	[−0.50, 0.16]		
	Measure type (EF)	Direct	3	−0.164	[−0.32, 0.00]	0.214	0.643
		Indirect	6	−0.235	[−0.46, 0.02]		

Note: K = number of studies; r = correlation coefficient; CI = confidence interval; Qb = heterogeneity; *p* = probability.

## Data Availability

All data generated or analyzed during this study are included in this published article.
